# From amoeboid myosin to unique targeted medicines for a genetic cardiac disease

**DOI:** 10.3389/fphys.2024.1496569

**Published:** 2024-10-28

**Authors:** James A. Spudich

**Affiliations:** Department of Biochemistry, Stanford University School of Medicine, Stanford, CA, United States

**Keywords:** myosin, translational research, hypertrophic cardiomyopathy, mavacamten, aficamten

## Abstract

The importance of fundamental basic research in the quest for much needed clinical treatments is a story that constantly must be retold. Funding of basic science in the USA by the National Institutes of Health and other agencies is provided under the assumption that fundamental research eventually will lead to improvements in healthcare worldwide. Understanding how basic research is connected to clinical developments is important, but just part of the story. Many basic science discoveries never see the light of day in a clinical setting because academic scientists are not interested in or do not have the inclination and/or support for entering the world of biotechnology. Even if the interest and inclination are there, often the unknowns about how to enter that world inhibit taking the initial step. Young investigators often ask me how I incorporated biotech opportunities into my otherwise purely academic research endeavors. Here I tell the story of the foundational basic science and early events of my career that led to forming the biotech companies responsible for the development of unique cardiac drugs, including mavacamten, a first in class human β-cardiac myosin inhibitor that is changing the lives of hypertrophic cardiomyopathy patients.

## Introduction

The Prix Galien USA award, America’s preeminent prize acknowledging the leading-edge of scientific advances in life sciences, was given for mavacamten (Mava) as the best biotechnology product of 2023. Mava was developed by MyoKardia, Inc., founded in 2012, only 11 years prior to this recognition, for treatment of hypertrophic cardiomyopathy (HCM). It is a β-cardiac myosin inhibitor. After highly successful phase 3 clinical trials, MyoKardia was acquired by Bristol Myers Squibb in 2020, and in 2022 they received approval from the U.S. Food and Drug Administration (FDA) and launched Mava as Camzyos™ for the treatment of adults with symptomatic hypertrophic obstructive cardiomyopathy (HOCM) to improve functional capacity and symptoms. HOCM is a subcategory of HCM that results from asymmetric septal hypertrophy, causing outflow obstruction of the left ventricle. Camzyos is the first and only FDA-approved allosteric and reversible inhibitor selective for cardiac myosin that targets the underlying pathophysiology of HOCM.

Hypertrophic cardiomyopathy (HCM) is a genetic disease that affects 1 in 500 to 1 in 200 individuals (Semsarian et al., 2015; Virani et al., 2020). It is characterized by left ventricular hypertrophy in the absence of predisposing conditions and leads to decreased volume of the left ventricular chamber. HCM is associated with mutations in genes encoding various sarcomeric proteins (Seidman and Seidman, 2001), but most mutations occur in either *MYH7* or *MYBPC3*, encoding human β-cardiac myosin heavy chain and cardiac myosin binding protein-C (MyBP-C), respectively (Alfares et al., 2015).

The physiology of the heart is fascinating and in recent times has been explored using new imaging modalities to reveal fundamental characteristics of normal and pathological conditions (Fukuda et al., 2021; Fukuda et al., 2023). HCM mutations result in hyperdynamic cardiovascular physiology (hypercontractility) that is often seen as a supranormal ejection fraction (EF) on echocardiograms even before hypertrophy is manifest (Ho et al., 2002; Captur et al., 2014; Haland et al., 2016).

The mutations in the human β-cardiac myosin heavy chain gene *MYH7* are missense mutations, found throughout the molecule but concentrated in the motor domain. As described later in this article, they primarily lead to de-stabilization of a myosin *OFF-state* that exists in the sarcomere, which results in more myosin molecules being available for interaction with actin and hypercontractility of the heart (Spudich et al., 2024). Mutations in *MYBPC3* primarily cause truncations of the protein, which leads to haploinsufficiency (Marston et al., 2009; van Dijk et al., 2009; Harris et al., 2011). Since one role of myosin binding protein C is thought to be stabilization of the myosin *OFF-state* (Spudich, 2015), haploinsufficiency of myosin binding protein C would also result in more myosin heads available for interaction with actin, again contributing to hypercontractility of the heart. Thus, a treatment for HCM could be a small molecule inhibitor of human β-cardiac myosin that reduces the contractility of the heart back to normal levels, potentially eliminating the signals that eventually cause hypertrophy of the heart and associated aspects of heart failure. Such an inhibitor is mavacamten (Mava), developed by MyoKardia and marketed by BMS as Camzyos™. And aficamten, a promising second-in-class cardiac myosin inhibitor being developed by Cytokinetics, is in Phase 3 clinical trials (Maron et al., 2024).

As a co-founder of MyoKardia and a Professor at Stanford who has studied myosins and other cytoskeletal proteins of muscle and nonmuscle cells for more than 5 decades, I am often asked, “how did your academic work lead you to enter the world of biotechnology, and how did that lead to the development of mavacamten.” The story is a classic bench-to-bedside narrative that started with fundamental biochemistry and biophysics and mechanistic understandings that paved the way to the development of the small molecule drug. Seldom does one learn how such developments were initiated, advanced, and brought to fruition. The Mava story involves many individuals, all contributing in important ways, and the story can be told from different perspectives depending on the individual’s role. Such stories are often told by Chief Executive Officers (CEOs) since they drive the business side of the relevant companies, essential for bringing a drug to market. The stories seldom reach back to the foundational basic science that made it all possible and early events that led to the formation of the company. Here, I tell the story of Mava from my perspective as a Co-Founder of MyoKardia and contributor to the foundational basic science that led to a detailed molecular understanding of how myosin works as a molecular motor.

My focus here on the reductionist approach should not be interpreted as a lack of appreciation of the invaluable and necessary contributions of cell- and organ-based studies both to the understanding of how muscle works (for review, see (Rall, 2014)) and to the development of Mava as a drug. As described in a recent review on Mava by Suman Nag and colleagues (Nag et al., 2023), beyond purified protein studies, studies with ventricular fibers isolated from mouse hearts, mouse cardiomyocytes, guinea pig cardiomyocytes harboring HCM mutations, human induced pluripotent stem cell-derived cardiomyocytes, engineered human heart tissue, and human ventricular tissues harboring missense myosin HCM mutations all provided critical information as Mava was being developed and tested. For example, in studies using isolated electrically-paced myocytes, mavacamten reduced sarcomere shortening and accelerated sarcomere relaxation (Toepfer et al., 2019; Sparrow et al., 2020). Another example is the demonstration that Mava preserves length-dependent contractility and improves diastolic function in human engineered heart tissue (Sewanan et al., 2021). Following successful tests with muscle fibers and cell systems, animal studies were necessary to see how well the *in vitro* and cellular model systems translate to the physiology of an animal. For example, in mice harboring heterozygous HCM pathogenic mutations in the α-myosin heavy chain, Eric Green and colleagues (Green et al., 2016) demonstrated that chronic administration of Mava suppressed the development of ventricular hypertrophy, cardiomyocyte disarray, and myocardial fibrosis. Furthermore, Mava treatment reversed hypertrophy after it occurred. Since the myosin isoform in mice (α-myosin) is different from humans (β-myosin), the effects of myosin inhibition by Mava were also studied in cats, which uses β-myosin for ventricular contraction. In the cat HOCM model, Mava caused reductions in both fractional shortening and inducible left ventricular outflow tract (LVOT) obstruction (Stern et al., 2016). Other animal studies were carried out with mini-pigs and dogs (Nag et al., 2023), which showed sufficiently encouraging results to warrant clinical studies with humans. Extensive clinical studies were pursued – phase 1 trials to test for toxicity, phase 2 trials to look for signs of efficacy, and then phase 3 trials to test a large number of patients for efficacy along a number of important parameters. The highlights from these clinical trials and references to many articles discussing the clinical benefits of Mava in detail can be found in (Nag et al., 2023). The bottom line is that changes in the functional parameters observed in patients with Mava treatment aligned well with those from preclinical studies, including those from *in vitro* molecular studies, which provided a common mechanistic basis for the observed functional changes predicted by the fundamental biochemical, biophysical and structural studies with purified proteins. That is not always observed in drug discovery, and I believe is a testament in the case of the Mava story to how deep the molecular understanding of the muscle contractile system is. So, base your new biotech initiative on a very solid foundation of molecular characterization of your biological system of interest.

I want to emphasize that this is not a review of the decades of muscle research carried out by the many basic scientists who made pivotal contributions, using biochemistry, biophysics, and cell- and organ-based physiology, throughout the decades and centuries since the original discovery of muscle myosin by the German physiologist Wilhelm Kühne in 1864 (for review, see (Szent-Gyorgyi, 2004)). To describe all those contributions would take multiple book volumes, an excellent one of which was written by Jack Rall in 2014 (Rall, 2014). Rather, this article is focused on answering the question I am often asked – how the work from my academic laboratory led me to enter the world of biotechnology, and how did that lead to the development of Mava. This is a story of how interdisciplinary research can drive the discovery of a protein target never pursued before for treatment of a devastating disease for which no prior therapeutics were available. In telling the story, I give the background and expertise of many of the key players involved to illustrate the importance of networking and how it takes many different clinicians and scientists, with different expertise, to go from the molecular biology of an important protein to the development of clinically active inhibitors.

## Myosin takes ∼10-nm steps along actin filaments by swinging its “lever arm” from a prestroke to a poststroke position every turn of the ATPase cycle

My early training was in chemistry and physics at the University of Illinois, Urbana-Champaign, in biochemistry and genetics at Stanford University, and in structural biology at the Medical Research Council Laboratory of Molecular Biology in Cambridge, England. With that background, since 1971 my laboratory has used interdisciplinary approaches to understand how the molecular motor myosin works and what roles it carries out in muscle and nonmuscle cells.

**FIGURE 1:**
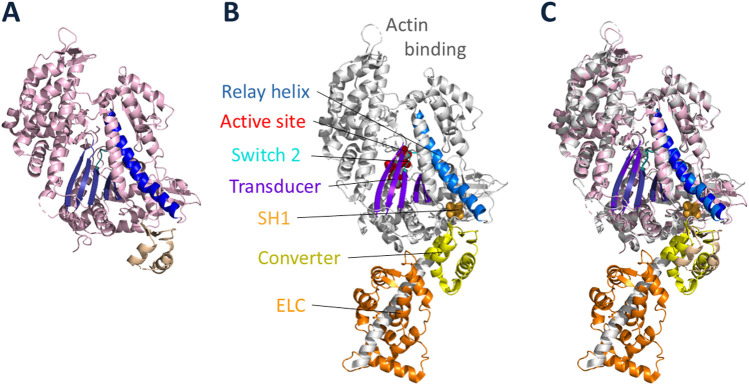
High-resolution structures of *Dictyostelium* myosin-II and cardiac myosin head domains. **(A)** *Dictyostelium* myosin-II globular head in the prestroke state, ending after the converter domain (PDB code: 1W9L, (Coureux, Sweeney and Houdusse, unpublished)). **(B)** Cardiac myosin globular head in the prestroke state (PDB code: 8QYQ (Auguin et al., 2024),) showing the heavy chain and associated ELC (orange). The heavy chain is largely shown in light grey with key elements highlighted: actin binding region; relay helix (blue); active site of nucleotide binding (red); switch 2 loop (cyan); transducer (purple); reactive cysteine residue called SH1 (light orange); converter (yellow). **(C)** overlay of *Dictyostelium* and cardiac globular head domains.

To achieve these goals, it is essential to reconstitute the functions of interest from purified proteins (Spudich, 2024). Thus, I sought an organism that could be easily grown in large quantities for biochemical studies and had the potential for using a molecular genetic approach to understand functions of the proteins of interest. After exploring multiple model eukaryotic cell types, *Dictyostelium discoideum* proved to be the organism of choice (Spudich, 2012). In the first few years of my laboratory, my postdoctoral fellow Margaret Clarke discovered and characterized a myosin-II in *Dictyostelium* (Clarke and Spudich, 1974), one of the early discoveries of myosin in nonmuscle cells (Hatano and Tazawa, 1968; Adelman and Taylor, 1969a; Adelman and Taylor, 1969b; Pollard and Korn, 1973a; Pollard and Korn, 1973b). *Dictyostelium* myosin-II is very similar to mammalian muscle myosin-IIs. The tertiary structure of its globular head domain (Figure 1A) is nearly identical to mammalian myosins (Figures 1B,C), which is true of all known myosins. The critical elements of all myosin head domains include an actin-binding region, a relay helix (Figure 1B, blue) which connects the nucleotide active site (red) to the converter (yellow), a switch-2 helix (cyan) which is involved in ATP hydrolysis and product release from the active site, a transducer (purple) made up of a β-pleated sheet which is thought to be primarily responsible for communication between actin binding and changes in the nucleotide active site, and a light chain binding region which is discussed in detail below. Many myosins also have a reactive cysteine residue called SH1 (Figure 1B, orange) which is very near the converter and can be used to attach probes to the myosin head. Given this similarity amongst all myosins, detailed mechanistic studies with *Dictyostelium* myosin are relevant to how cardiac myosin converts the chemical energy of ATP hydrolysis into mechanical movement, and my story starts with Margaret Clarke’s discovery of *Dictyostelium* myosin-II in 1974.

**FIGURE 2:**
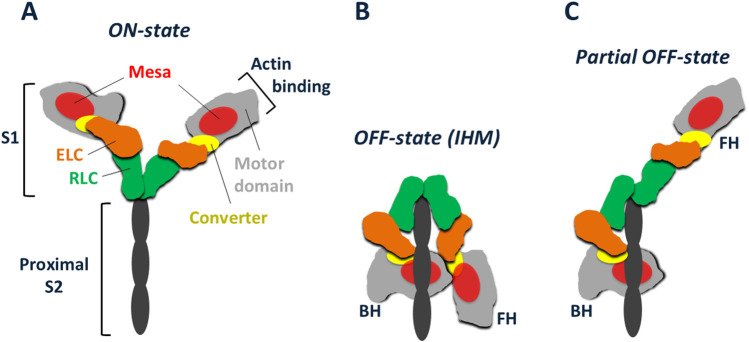
Schematic representation of cardiac myosin in various conformations. **(A)** Cardiac myosin with its S2 tail truncated to its proximal portion. The S1 heads are shown in their *ON-state*, free to interact with actin. Each S1 consists of a globular heavy chain and two light chains. The globular motor domain (light grey) with its relatively flat mesa surface (red) has the converter (yellow) at its C-terminal end. The essential light chain (ELC, orange) and regulatory light chain (RLC, green) are bound to an α-helix of the heavy chain extending from the converter. Further extension of the heavy chain α-helix gives rise to the S2 coiled-coil, the proximal portion of which is shown (black). **(B)** The IHM *OFF-state* of cardiac myosin, showing the interaction of the blocked head (BH) with both the proximal S2 tail and the free head (FH). Modeled after the high-resolution human β-cardiac IHM structure (Grinzato et al., 2023). **(C)** A possible *partial OFF-state* with the BH still unable to interact with actin due to its binding to the proximal tail, while the FH has become free to interact with actin.

Myosin-IIs have two globular heads, each known as Subfragment-1 (S1) of myosin, followed by a coiled-coil tail (Figure 2A). The S1 consists of a motor domain (Figure 2A, light grey) ending in its converter (Figures 1, 2A, yellow), followed by a light chain binding domain with one essential light chain (ELC, orange) and one regulatory light chain (RLC, green). Not shown in Figure 2A, the tail is divided into two domains, Subfragment-2 (S2), which follows directly after the S1 heads, and light meromyosin (LMM), which extends to the C-terminus of the molecule. LMM self assembles and forms the shaft of the muscle thick filament, while S2 is free to move away from the thick filament and bring the myosin heads in contact with the actin filaments. Margaret showed that *Dictyostelium* myosin-II has all the fundamental properties of mammalian muscle myosins, including the ability to assemble into bipolar thick filaments.

Later my graduate student Arturo De Lozanne disrupted the single copy *Dictyostelium* myosin II gene using molecular genetic approaches and discovered that high-efficiency homologous recombination occurs in this organism (De Lozanne and Spudich, 1987), a surprise at the time to the scientific community. Arturo’s work provided the first genetic proof that myosin II is needed for cytokinesis of cells in suspension but, surprisingly, not for general cell migration.

We then created a *Dictyostelium* myosin-II null cell by knocking out the gene (Manstein et al., 1989). While not able to divide in suspension, the myosin-II null cells could be kept alive by growing them on a surface and allowing them to “divide” by a process we named *traction-mediated cytofission.* We were able to rescue cytokinesis in suspension by expressing a GFP-tagged version of the cell’s normal myosin-II off a plasmid and to track and quantify the localization of the fluorescent myosin to the furrow region just prior to cell division (Supplementary Movie 1). Using *Dictyostelium*, we also carried out extensive studies regarding the roles of myosin-II in nonmuscle cells, and the organism proved critical for understanding how myosin works in muscle contraction as well, as described below.

Interestingly, prior to the mid-1980s, there was ongoing debate in the muscle field regarding Hugh Huxley’s swinging crossbridge hypothesis for muscle contraction and nonmuscle cell movements, in which he proposed that the S1 rotated as a rigid body while bound to actin (Huxley, 1969). Given the dimension of the S1 (about 15 nm long), rotation of 70° or so would provide ∼10 nm of movement of the actin for each round of the actin-activated ATPase cycle. Between 1969 and 1983, Huxley’s hypothesis was falling out of favor for two reasons. First, experiments from the laboratories of Roger Cooke and Dave Thomas using probes bound to the SH1 cysteine residue in the myosin S1 globular head domain in skinned muscle fibers failed to reveal the expected rotation of the heads during contraction (Thomas and Cooke, 1980). These results prompted a new model of contraction that involved changes in dimension of the myosin tail (Ueno and Harrington, 1981; Harrington et al., 1983). Second, experiments from Toshio Yanagida’s laboratory suggested that the distance a single actin filament moves upon interacting with a myosin molecule during one round of ATP hydrolysis (the step size) could be ∼60 nm (Yanagida et al., 1985) or larger (Yanagida et al., 2000), much greater than the ∼10 nm step size expected by Huxley’s swinging cross bridge model. Consequently, a biased Brownian ratchet model was proposed in which the myosin head thermally diffuses along an actin filament according to asymmetric potentials (Yanagida and Iwane, 2000; Yanagida et al., 2000). What was missing were quantitative *in vitro* assays with reconstituted purified actin and myosin for measuring and quantitating the functions of interest – movement and force production (Spudich, 2024).

During the decade 1985–1995, my laboratory developed two assays essential to measure these functions of interest using purified proteins – first, an *in vitro* motility assay that allowed us to measure velocity of actin moving across a lawn of myosin molecules (Kron et al., 1985; Kron and Spudich, 1986) (Supplementary Movie 2), and second, a laser trap assay that allowed us to measure the step size and amount of force produced when one myosin molecule interacts with a single actin filament (Finer et al., 1994). For a detailed historical description of the development of these assays, see (Spudich, 2024). With these *in vitro* assays, we were able to resolve the results that were not compatible with Huxley’s swinging crossbridge model.

The power of having developed the *in vitro* motility assay was made immediately apparent by my postdoctoral fellow Yoko Toyoshima who showed that S1 is all one needs to drive the movement of actin filaments across a lawn of myosin (Toyoshima et al., 1987). This pivotal experiment showed that S1 is the motor domain of myosin and ruled out other hypotheses.

**FIGURE 3:**
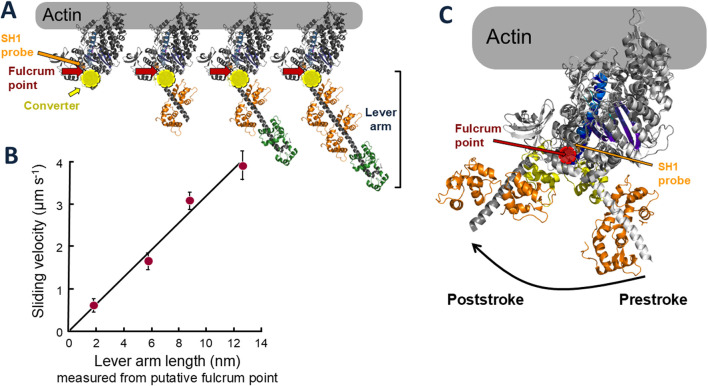
Lever arm constructs and sliding velocities of altered and wild type myosins. **(A)** Models of molecularly engineered S1 heads with 0, 1, 2 (wild type), and 3 light chains bound. A typical prestroke state myosin S1 head was used [PDB code: 8ACT, Free Head (Grinzato et al., 2023)]. The site of the cysteine residue known as SH1 is shown by the orange arrow. The predicted fulcrum point of rotation of the lever arm from the data shown in **(B)** is marked by the dark red arrows, just above the converter (yellow). **(B)** Sliding velocity of actin filaments along myosin constructs shown in **(A)** *versus* lever arm length. Data, taken from (Uyeda et al., 1996), are representative of four independent experiments with different preparations of proteins over a period of a year. Bars indicate standard deviations. **(C)** Prestroke [PDB code: 8QYQ (Auguin et al., 2024)] and poststroke [PDB code: 6FSA (Robert-Paganin et al., 2018)] myosin head configurations emphasizing the SH1 position (light orange) in relation to the fulcrum point (red) of the lever arm swing.

Then in 1993 the X-ray high resolution crystal structure of S1 was described by Ivan Rayment and his colleagues (Rayment et al., 1993a), and the orientation of the S1 bound to actin (Rayment et al., 1993b; Schröder et al., 1993) showed that the light chain binding region is on the opposite end of the S1 domain from the actin binding site (Figure 1A). Shortly after the S1 structure was solved, my postdoctoral fellow Taro Uyeda took advantage of our *Dictyostelium* myosin-II null cell to engineer, express and purify full length *Dictyostelium* myosin-IIs having zero, one, two and three light chains bound (Figure 3A) and used the *in vitro* motility assay to show a linear relationship between the velocity of actin movement on myosin-coated surfaces and the length of the light chain binding region (Figure 3B) (Uyeda et al., 1996). Note the globular head with its converter but without light chains moved at ∼0.5 μm s^−1^, showing that this is the motor domain, not the entire S1 head. The light chain binding region serves to amplify the velocity by increasing the length of the lever arm. Extrapolation of the best fit line in Figure 3B to zero velocity defined the position of the fulcrum point for the lever arm swing, which is just above the converter in Figure 3A. Thus, for wild type myosin, the lever arm includes the two light chains and the converter domain and is ∼10 nm long. These were compelling data to modify Hugh Huxley’s swinging crossbridge model, where the S1 head rotated as a rigid body, to a “swinging lever arm” model, where the main portion of the S1 does not change its orientation during contraction. This fixed portion contains the SH1 sulfhydryl group that Cooke and Thomas had labeled with an orientation-sensitive probe (Thomas and Cooke, 1980) (orange arrow, Figure 3A), which explains why they failed to see the rotation expected by Huxley’s swinging crossbridge hypothesis. SH1 is just above the predicted fulcrum point of rotation shown by the dark red arrow in Figure 3A. Later, high-resolution structures of a poststroke state confirmed the fulcrum point location (Figure 3C) (for reviews, see (Holmes, 1997; Geeves and Holmes, 1999; Sweeney and Houdusse, 2010a)), as did studies from David Warshaw and his colleagues who measured the displacement of actin using the dual-beam laser trap as a function of lever arm length of smooth muscle heavy meromyosin (HMM, a two-headed truncated form of myosin missing the C-terminal portion of its coiled-coil tail involved in thick filament formation) (Warshaw et al., 2000).

The next important step was resolving Yanagida’s large myosin step size issue. All experiments prior to 1994 were complicated by the fact that they involved an ensemble of myosin molecules, and assumptions were required to estimate the step size a single myosin molecule takes. Then, in 1994, Jeff Finer and Bob Simmons in my lab took our *in vitro* motility assay to the single molecule level using a dual beam laser trap system (Finer et al., 1994). When a single myosin molecule was observed to go through one cycle of interaction with actin using the laser trap, a step size of ∼10 nm was observed, consistent with the swinging lever arm model. This model was then confirmed by a multitude of studies from my laboratory and others using the processive motors myosin V and myosin VI [for reviews, see (Sweeney and Houdusse, 2004; Sellers and Veigel, 2006; Trybus, 2008; Sweeney and Houdusse, 2010b; Spudich and Sivaramakrishnan, 2010)], and decades of controversy regarding the mechanism of energy transduction by myosin were resolved. Also, the tools were now available to explore the effects of myosin mutations on the fundamental parameters that determine the power output of the motor. *Dictyostelium* offered a great opportunity in this regard since we had a *Dictyostelium* myosin-II null cell that could be transformed with mutant forms of the myosin to check for both rescue of myosin-dependent cell behaviors and changes in the fundamental biochemical and biophysical properties of purified mutant myosin.

## A prologue to detailed molecular studies of missense mutations that occur in β-cardiac myosin in HCM patients involved random mutagenesis of the motor domain of *Dictyostelium* myosin-II

**FIGURE 4:**
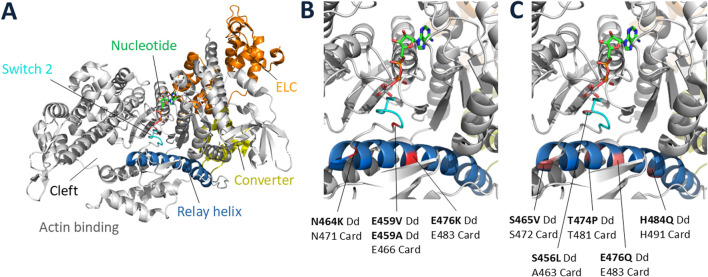
Locations of missense mutations in *Dictyostelium* myosin-II. **(A)** Full view of a typical myosin globular head domain in the prestroke state [PDB code: 5N69 (Planelles-Herrero et al., 2017)] showing the relay helix and switch 2 regions spanning *Dictyostelium* myosin-II residues 454–486, in which 21 random point mutations were created. Of those 21 mutant genes, 15 contained single amino acid substitutions, and the others contained double or triple amino acid substitutions. The 15 single-residue mutant myosins were classified as WT-like if they were able to fully rescue the null-cell behavioral defects (growth in suspension and development into fruiting bodies): 1455V, F461Y, K462N, Y473H, Y473S, F481V (not shown in Figure). **(B)** Blow-up of key elements showing the positions of the 4 null class mutations (failed to rescue null phenotypes): E459V, E459A, N464K, E476K. The corresponding positions in human β-cardiac myosin are indicated below the *Dictyostelium* residue numbers. **(C)** Blow-up of key elements showing the positions of the 5 intermediate class mutations (partially rescued null phenotypes): S456L, S465V, T474P, E476Q, H484Q. The corresponding positions in human β-cardiac myosin are indicated below the *Dictyostelium* residue numbers. The S456L mutation was originally classified as WT-like (Ruppel and Spudich, 1996a), but was later shown to actually be an intermediate class mutation (Murphy et al., 2001).

In what was a prologue to detailed molecular studies we began 15 years later of missense mutations that occur in β-cardiac myosin in HCM patients, Kathy Ruppel, an MD/PhD student in my lab, used random mutagenesis to create 15 single-residue missense mutant myosins in a highly conserved region of the motor domain of *Dictyostelium* myosin II. Kathy transformed the *Dictyostelium* myosin null cell with each of these to classify these missense mutations into three distinct groups based on their ability to complement myosin null cell phenotypes (loss of ability to divide in suspension and loss of ability to differentiate into fruiting bodies): wild type, intermediate, and null (Ruppel and Spudich, 1996a; Ruppel and Spudich, 1996b). Biochemical analysis of the mutated myosins also revealed three classes of mutants that correlated well with the phenotypic classifications. Myosin mutants that behaved like WT myosin in that they were able to fully rescue the cellular defects of the myosin null cell and had normal activity as purified proteins were all minor residue changes (Figure 4 legend). The mutated myosins that were not fully functional showed defects ranging from ATP non-hydrolyzers to myosins whose enzymatic and mechanical properties are uncoupled. Placing the mutations onto the three-dimensional structure of myosin showed that the mutated region consisted of the relay helix and switch 2 (Figure 4), revealing that this region of myosin plays a key role in transduction of chemical energy to mechanical displacement. The intermediate class S456L mutation, which lay in the middle of switch 2, proved particularly interesting in that it uncoupled ATPase activity from motility and resulted in a shortened step size (Murphy et al., 2001).

These were early experiments characterizing the effects of missense mutations on myosin function, only doable with the *Dictyostelium* system at that time. We would later translate those studies to human β-cardiac myosin, where the mutations are of serious clinical significance.

## My first engagement in biotechnology was as a member of the discovery board of SmithKline Beecham

By 1994, uncertainties about the fundamentals of how myosin transduces the chemical energy of ATP hydrolysis into mechanical movement were resolved, and the molecular basis of muscle contraction and nonmuscle cell movements, changes in cell shape, and intracellular trafficking were reasonably well understood (Hartman et al., 2011; Houdusse, 2021). At the time, I did not contemplate becoming involved in the world of biotechnology.

Then, in 1995 I was invited to join the Discovery Board of SmithKline Beecham, now Glaxo SmithKline. Twice a year the members of the Discovery Board listened to the many programs going on at SmithKline Beecham, and we were then asked to present our ideas of new directions they might consider. As a cytoskeletal biochemist and biophysicist, I asked why no one in the industry was targeting the cytoskeleton, which by that time was well characterized by the academic cytoskeletal community, and the cytoskeleton is often the downstream effector that many current upstream drugs eventually impact. Classic examples of such upstream drugs act on membrane receptors that indirectly regulate the contractile properties of the heart. Why not target the heart’s sarcomeric cytoskeletal proteins directly? By targeting those downstream proteins, one could obviate the pleiotropic effects caused by changing Ca^2+^ levels and phosphorylation patterns that result from current upstream heart-related drugs. I learned a great deal about the pharmaceutical world at those meetings and continued to recommend targeting cytoskeletal proteins for both cardiology and oncology. It was clear to me after 2 years on the Discovery Board, however, that launching a program developing therapeutics against cytoskeletal targets was not on their agenda.

Consequently, in 1997, I contacted my friend and colleague Ron Vale at UCSF, who had discovered the kinesin family of molecular motors in 1985 (Vale et al., 1985), and suggested we get together and talk about whether we should start a biotech company based on the cytoskeletal target platform. Ron said my call was very timely because he had already been talking with Larry Goldstein, another key player in kinesin biology (Yang et al., 1989; Vale and Goldstein, 1990; Goldstein, 1991), about the same idea. Conveniently, Larry had moved from his tenured faculty position at Harvard University in 1993 to UCSD in Southern California. This meant it was easy for him to fly up to SFO where the three of us met monthly at the United Airlines Red Carpet Club to discuss what scientific programs our proposed new biotech company would undertake. Two concrete programs emerged. One in oncology, with the concept that it would be much more specific and potentially less toxic to target mitotic kinesins to kill cancer cells rather than using drugs such as Taxol which interfere with microtubule function. Microtubule agents remain important oncology therapeutics since they are very effective, but toxicity is difficult to eliminate because microtubules are essential for many biological functions in nearly every cell in every organ of the body. We posited that the right kinesin inhibitor might be much better tolerated. The second obvious direction was for a very different clinical need, cardiac diseases. We imagined increasing power output of a diseased heart by targeting and activating the sarcomeric protein contractile system itself. The idea was to target the downstream effectors, eliminating the negative pleiotropic effects of targeting upstream cell components such as membrane signaling systems, typical of existing cardiac drugs.

## Cytokinetics, the first biotech company to target cytoskeletal molecular motors, was founded in 1998

Anna Spudich, who received a PhD from Stanford for her work on the actin cytoskeleton of the sea urchin *Strongylocentrotus purpuratus*, was collaborating with me at the time of the Red Carpet Club discussions that Ron, Larry, and I were having. One evening over dinner, she pointed out that if the three of us wanted to start a biotech company, we needed to find someone to devote 100% time to lead the effort, and that person needed to be smart, young and energetic, willing to take risks, a quick learner, have a natural ability to energize people, and have a sense for business. In addition, this leader of the effort should have a good understanding of the cytoskeletal field and its potential importance in medicine. She knew just the right person, James Sabry. Anna knew James well because they sat across from one another in my laboratory in the Beckman Center at Stanford University School of Medicine. James had an MD degree from Queens University Medical School in Kingston, Ontario, and a PhD from UCSF working with Marc Kirschner. He already had a part time clinical position at UCSF but wanted to further his basic science training by carrying out postdoctoral work in my laboratory. After the discussion with Anna the previous evening, the next morning I approached James with Anna’s idea, just as he was about to leave for the airport to fly to Boston to probably accept a faculty and physician position at Harvard’s Brigham and Women’s hospital, with his lab specializing in neurobiology. When he returned from his trip a few days later, he said “I told them no. When can we start this biotech company.” This was a bold step, but James never looked back. He became a 4^th^ Founder, joined the Red Carpet Club meetings, and then led our new company, Cytokinetics, founded in 1998, as CEO for the first decade of its existence. He was perfect for the position!

Our early days of fundraising for Cytokinetics were discouraging but not atypical for a group of academic basic scientists wanting to start a biotech company with an entirely new focus. Comments from the first venture capitalists (VCs) we spoke with included, “what are you guys smoking?” and “major pharmaceutical companies have oncology and cardiac disease high on their list. You are naïve to think you can compete.” Then one evening in Dortmund, Germany, a meeting with Greg Petsko, a biochemist at Brandeis at the time, provided the contact we needed in the VC community. Greg and I were having a glass of wine after a day of listening to presentations by faculty of the Dortmund Max Planck Institut headed by Roger Goody. Greg and I were both on their Advisory Board. Greg was involved in biotechnology and, upon hearing my story about our Cytokinetics fund raising attempts, he recommended we talk to Steve Dow at Sevin Rosen Funds in Palo Alto and made the necessary introduction. Steve had provided funding for one of Greg’s adventures. When I got back to Stanford, my Co-Founders and I met with Steve Dow for lunch in Palo Alto. Steve could see we were totally naïve about the biotech industry but saw that we had something unique to offer and that the four Founders made a good team – he called us “the four amigos.”

Steve reached out to Grant Heidrich at Mayfield Fund who met with us and agreed to join. Roy Vagelos and I knew each other from our associations with the American Heart Association, and he agreed to be a Round A investor. Suddenly, we had two top VCs and a world-renowned leader of science, biotechnology and the pharmaceutical industry, Roy Vagelos, advising us on how to start a successful new biotech adventure. James Sabry, as CEO, and I represented the Co-Founders on the Board of Directors. I took a year leave of absence from Stanford and spent the first year of the company working with our small team at Cytokinetics in South San Francisco.

Critical for success of any new initiative is hiring the right people, and our initial hires were all stellar. Our first hire was Fady Malik, an MD-PhD student at UCSF who did his PhD work with Ron Vale and did his clinical training in cardiology. Fady was perfect for our cardiac muscle program and remains at Cytokinetics as a key leader 25 years later, now as Executive Vice President of Research and Development. Jeff Finer, a Stanford MD-PhD student from my laboratory, left his Harvard residency program to join the company. As mentioned above, Jeff’s PhD thesis was developing the laser trap technology in my laboratory for measuring nanometer movements and picoNewton forces of single myosin molecules, for which he is well known. Other key players also came from the Co-Founder’s labs, Roman Sakowitz from Larry Goldstein’s laboratory and Jim Hartman from Ron Vale’s. Robert Blum, the current President and CEO of Cytokinetics, was also a very early recruit from his position as Director of Marketing at COR Therapeutics, joining Cytokinetics in July 1998 as Vice President of Business Development. Robert now leads the company as President and Chief Executive Officer.

As Roy Vagelos always emphasized, drug discovery is all about chemistry. Early chemists at Cytokinetics were Evan Lewis and Todd Tochimoto, who worked under the direction of Jeff Finer and consultant Jack Chabala. Chemist Gustav Bergnes joined Cytokinetics in December 1999 as a Scientist III after 7 years in the biotech industry, and chemist Bradley (Brad) Morgan, currently Senior Vice President of Research and Non-clinical Development at Cytokinetics, joined the company in 2002 after holding several scientific and management positions at Pfizer Global Research. All these individuals plus others contributed in pivotal ways in the early days of the company, and Robert Blum, Fady Malik, Jim Hartman, and Brad Morgan are among early hires who have stayed with Cytokinetics throughout its history, devoted to its current mission of developing potential medicines that improve the health span of people living with cardiovascular and neuromuscular diseases of impaired muscle function.

While Cytokinetics began as a two-program company, one in oncology targeting kinesins and the other in muscle diseases targeting the muscle actin-myosin-based contractile system, in 2008 the company discontinued research activities in oncology and realigned with a focus to muscle biology. Since then, the company has built a robust pipeline of sarcomere-directed therapies with promise for people living with diseases characterized by muscle weakness, loss and dysfunction. A key program for Cytokinetics has been treatment of heart failure, for which they developed omecamtiv mecarbil (OM) (Morgan et al., 2010). OM binds directly to myosin and activates the myosin-based contractile system of the heart.

## Hypertrophic cardiomyopathy is a genetic disease that causes hypercontractility of the heart

While Cytokinetics was screening for small molecule activators of cardiac myosin, many inhibitors were found and temporarily put on the shelf. Cytokinetics considered developing their myosin inhibitors for treatment of hypertrophic cardiomyopathy (HCM), since HCM is a genetic disease that leads to hypercontractility of the heart. An inhibitor that would bring contractility down to normal levels would possibly obviate what was likely hypercontractility-induced hypertrophy and other deleterious syndromes characteristic of the disease. The frequency of HCM in the population, while significant, is much smaller than the general heart failure population that OM was designed to treat. Reasonably, the company decided it would be better to use resources to diversify into other areas of muscle physiology and began programs in skeletal muscle and smooth muscle. Furthermore, and importantly, at the time there was no clear evidence whether the hypercontractility seen in patients carrying an HCM mutation in β-cardiac myosin had its origins in an increase in power output by the mutated myosin itself. If so, it is hard to think of a more direct therapeutic program than targeting the very protein that is causing the hypercontractility with a small molecule that brings the power output of the myosin back to normal levels. *But do these β-cardiac myosin mutations result in a myosin that produces higher output than normal?* This would require the ability to express and purify functional human β-cardiac myosin in the laboratory but attempts to do so had all failed.

As a substitute for human β-cardiac myosin, David Warshaw and his colleagues, in collaboration with Kricket and Jon Seidman, used constructs of mouse α-cardiac myosin carrying HCM mutations (Tyska et al., 2000). They showed, for example, that α-cardiac mouse myosin containing the R403Q mutation had both an increase in velocity and in ATPase activity. These were important early studies to characterize the effects of HCM mutations on myosin function. But then Susan Lowey and coworkers showed that the effects of the R403Q mutation in mouse cardiac myosin depended on the isoform into which the mutation was introduced (Lowey et al., 2008). In mouse α-cardiac myosin, they confirmed an increase in both ATPase activity and velocity, but in mouse β-cardiac myosin there was no significant change in the velocity and a slight decrease in the ATPase activity. If human β-cardiac myosin behaved like the mouse β-cardiac myosin, one would have to question the hypothesis that the hypercontractility seen in patients carrying an HCM mutation in β-cardiac myosin had its origins in an increase in power output from the mutated myosin itself. Note that mouse β-cardiac myosin has more than 30 residue differences compared with human β-cardiac myosin, and in HCM one is looking for changes due to a single residue change. It is therefore difficult to translate results from mice to man. *Thus, it was imperative that one study human β-cardiac myosin itself to ascertain the true effects of HCM mutations on cardiac myosin’s power output.*

I had wanted for some time to extend our earlier work on missense mutations in *Dictyostelium* myosin to study the effects of HCM mutations in human β-cardiac myosin, where the mutations have serious clinical consequences. The goal was to quantitate the biochemical and biophysical properties of human β-cardiac myosin carrying HCM mutations using our battery of interdisciplinary tools, including methods we had developed for measuring the velocity of movement of purified actin along purified myosin molecules using our *in vitro* motility assay, as well as for measuring the intrinsic force of myosin using a dual-beam laser trap. The problem was we only wanted to initiate this long-term program if we could study the human β-cardiac myosin isoform, but as already mentioned no one was able to express functional human β-cardiac myosin in any conventional expression system.

A resolution to the problem came from my long-time collaborator and friend Leslie Leinwand from the University of Colorado, Boulder. Leslie called to say that she had used a mouse myogenic cell line C2C12 to express a mammalian myosin that had good actin-activated ATPase activity and asked if we could check out its activities in our assays. Hans Warrick in my lab showed that it was indeed active in our *in vitro* motility assay (Resnicow et al., 2010; Deacon et al., 2012), and suddenly the window of opportunity was opened to study human β-cardiac myosin mutations in purified expressed protein. The timing was perfect – Alex Dunn and Zev Bryant, postdocs in my lab, were completing what we considered to be the critical experiments on myosin V and myosin VI that solidified the swinging lever arm hypothesis as the mechanism of how myosin transduces the chemical energy of ATP hydrolysis into mechanical movement (Dunn and Spudich, 2007; Bryant et al., 2007). Remaining experiments would be dotting i’s and crossing t’s. Thus, in 2010 we completed the work we were doing on characterizing myosins V and VI and the entire laboratory focused on studying human β-cardiac myosin with well-authenticated HCM mutations that had been categorized as clearly causative of HCM. Pivotal for these studies was Kathy Ruppel’s accepting my offer and moving to Stanford from her faculty position at UCSF. Kathy joined my laboratory in the Department of Biochemistry at Stanford as a senior collaborator, with a faculty appointment in Pediatric Cardiology. As a former MD-PhD student in my lab who had extensive experience studying myosin structure and function with excellent clinical training at Stanford and then at Harvard in cardiology, she was perfect for this new adventure!

At that time, the expressed cardiac myosin available carried the mouse skeletal light chains. So, Kathy, working closely with the Leinwand lab, established the expression of human β-cardiac myosin containing the relevant human ventricular essential (ELC) and regulatory (RLC) light chains. The availability of a pure human β-cardiac myosin reconstituted with the relevant human cardiac ventricular light chains was a major step that allowed the study of the effects of HCM-causing mutations in the human β-cardiac myosin gene *MYH7* on the functions of interest that contribute to the hypercontractile state of the heart.

The two fundamental parameters of the heart that contribute to its power output (P) are velocity of contraction (v) and the force (F) the heart produces, since P = F^*^v. These are both set by the central properties of the myosin molecular motor, and both could be measured with purified proteins using a variety of biochemical and biophysical assays, including the *in vitro* motility assay and the single molecule laser trap assay. The force the heart produces is the ensemble force of all the independent myosin force generators. Thus, F_ensemble_ = F_intrinsic_ (t_s_/t_c_)N_a_, where F_intrinsic_ is the force produced by each myosin molecule, t_s_ is the strongly bound time of a myosin head to actin for one ATPase cycle, t_c_ is the overall cycle time, t_s_/t_c_ is the duty ratio or the fraction of heads bound and producing force at a particular time during contraction, and N_a_ is the total number myosin heads in the muscle accessible for interacting with actin. Three of these fundamental parameters are readily measured with assays we had developed: v is measured with the *in vitro* motility assay, F_intrinsic_ is measured with the laser trap, and t_c_ (1/k_cat_) is measured with a standard ATPase biochemical assay.

The first measurements with two HCM mutant forms of expressed and purified human β-cardiac myosin that we studied, R403Q and R453C, showed that indeed fundamental parameters were increased in the mutant myosins. R403Q showed a 25% increase in actin-activated ATPase activity and a 15% increase in velocity (Nag et al., 2015) compared to wild type human β-cardiac myosin, and R453C showed a 50% increase in F_intrinsic_ (Sommese et al., 2013). These results supported the hypothesis that hypercontractility seen in patients carrying an HCM mutation in β-cardiac myosin had its origins in an increase in power output from the mutated myosin itself.

## MyoKardia was launched in 2012 specifically to treat hypertrophic cardiomyopathy patients

It was at this time that I received a call from Charles Homcy, who I knew well from our time together as members of the Board of Directors of Cytokinetics. Charles, a cardiologist trained at Johns Hopkins, has considerable experience building and leading successful biotech companies, discovering and developing novel therapeutic approaches. For example, he co-founded and served as President and Chief Executive Officer of Portola Pharmaceuticals, Inc. In 2010, Charles was asked by the Boston-based venture capital firm Third Rock Ventures to join Third Rock and help them establish a San Francisco-based office. That was the purpose of his call with me. He asked if he could come to my office and get my thoughts about exciting new science going on at Stanford that might be ripe for starting new biotech companies, funded by Third Rock Ventures. We scheduled a 1-h meeting in which I described research going on in several of my colleagues’ laboratories, which I thought were worth exploring as potential new biotech startups. At the end of the conversation, he asked me what we were now doing in my laboratory. It had been a while since we talked, and he was surprised to hear that my lab was now entirely focused on studies of hypertrophic cardiomyopathy. We spent the rest of the afternoon going over our results and my excitement about being able to characterize the effects of HCM mutations at the molecular level. At the end of the day, Charles said, “Jim, you have to start another company!” I agreed to come to his offices in San Francisco to give a seminar to the Third Rock Ventures San Francisco group, which consisted of Charles and his young protégé Neil Kumar. Neil and I knew each other from Stanford, where he received his BS and MS in Chemical Engineering, 1997–2002. During those years, Neil was very interested in our development of laser trap technology for single-molecule analyses of the myosin family of molecular motors. Charles was mentoring him in the ways of the venture capital world. At the end of my seminar, they both said, “you have to start a company based on this.” A couple of months later, Kevin Starr, founding partner of Third Rock Ventures, was visiting from Boston, and I provided an update of my earlier seminar, now to an audience of three. Kevin said, “this looks like a wonderful beginning of a new biotechnology company based on hypertrophic cardiomyopathy.” Charles asked, “what should we call it,” and without thinking deeply about it, I said, “how about Myocardia, make that with a k, no, make it with a capital K,” and MyoKardia was tentatively given birth.

Third Rock suggested we add additional key Founders, and I suggested Leslie Leinwand, a collaborator in our various academic studies and an expert on the genetics and molecular physiology of inherited diseases of the heart. Leslie was trained in molecular biology, served as Director of the Cardiovascular Research Center at Albert Einstein College of Medicine, then chaired the Department of Molecular, Cellular, and Developmental Biology at the University of Colorado Boulder. She was not a newcomer to the biotech world, having been a Co-founder of Myogen.

There was a third meeting with a larger group from Third Rock Ventures at their Boston office, where I gave a third update of our work. Leslie Leinwand participated and Christine (Kricket) Seidman and Jon Seidman, well known for their work on the genetic basis of hypertrophic cardiomyopathy (Seidman and Seidman, 2001), were also invited. Kricket is Director of the Cardiovascular Genetics Center at Brigham and Women’s Hospital and Jon is a geneticist at Harvard Medical School. They are recognized for discovering the first genetic cause of congenital heart defects and are leaders in this important field. Shortly following that meeting, Leslie and I called Kricket and John inviting them to become Co-Founders of MyoKardia, and they immediately agreed. Leslie, Kricket, Jon and I complemented one another perfectly, and MyoKardia was incorporated in June of 2012 with $0.5 million seed funding from Third Rock Ventures. Three months later Third Rock established a Round A financing of $38 million. It was an awesome beginning.

To start the new company, in July 2012 we hired a Senior Vice President of Drug Discovery, Robert (Bob) McDowell, and a Senior Director of Biology, Hector Rodrigues, with Charles Homcy serving as acting CEO. Hector’s involvement in biotechnology began at Cytokinetics in 2001, where he was Scientist III, Biochemistry. In 2007 he moved to Arresto BioSciences as Head of Analytical Biochemistry, and in 2010 to Calithera Biosciences as Associate Director of Biochemistry. Prior to joining MyoKardia, Bob McDowell led drug discovery at 3-V Biosciences, advancing the company’s lead program into development. Before that he served as Vice President of Research at Sunesis Pharmaceuticals. He then led the structural chemistry group at Axys Pharmaceuticals. He assumed the position of Chief Scientific Officer at MyoKardia in 2017 and remained at MyoKardia until it was acquired by Bristol Myers Squib in 2020. Thus, Bob was instrumental and a key participant in the development of Mava from MyoKardia’s inception through the company’s entire existence.

To jump-start the science, we were fortunate that Robert Blum and the Cytokinetics Board of Directors agreed to have us incubate at Cytokinetics and to license several of their cardiac myosin inhibitor HITS from their compound library assembled from commercially available small molecules. Thus, by September 2012 we were up and running in space leased from Cytokinetics. Drug discovery is largely about using chemistry to alter such HITS through structure-activity-relationship (SAR) studies to search for a molecule that has the appropriate potency, selectivity, stability, as well as a host of other important characteristics to enable it to be a clinically relevant drug. One of those early HITS was chemically modified leading to R & S stereoisomers. The R isomer was inactive in the actin-activated cardiac myosin ATPase biochemical assay, but the S isomer (later named mavacamten) was an excellent inhibitor. Thus, MyoKardia had Mava before the end of 2012. Little did we know that a decade later the molecule which we had in hand within the first 6 months of incorporating the company would prove to be the clinical lead molecule, and then the very molecule that the FDA approved to be sold as Camzyos.

Throughout the first year, Charles Homcy was acting CEO, and he, with input from the Co-Founders, was searching for an appropriate person to lead the company going forward. We were determined to find someone with the same passion for our quest that Charles Homcy, the Co-Founders, and early hires had. After interviewing multiple candidates, Tassos Gianakakos was identified. Tassos has an interesting CV, with BSc degrees in chemical engineering and economics from MIT, an MSc in biotechnology from Northwestern University, and an MBA from Harvard Business School. Prior to joining MyoKardia, Tassos was Senior Vice President and Chief Business Officer at MAP Pharmaceuticals, and before that he led the formation of Codexis, Inc., a spin-off of Maxygen, where he was Director of Business Development. At Codexis, Tassos served as President and Senior Vice President of Business Development, and Global Head of Codexis’ Pharmaceuticals Business Unit.

I remember well Tassos’ interview with me in 2013. He showed deep interest in our goals, in the team we had already built, and the progress that we had made in a short time. His enthusiasm and vitality, combined with his prior training, made him our top candidate. Everyone agreed and Tassos took the position as CEO in late 2013. His remarkable business leadership and the company of several hundred individuals he built, with key individuals at every intersection of the company’s goals, were essential for Mava’s outstanding success story.

Key to Mava’s success was having a deep understanding of the actin-activated myosin system and the insight to go after targeting the downstream effector, minimizing pleiotropic effects that often lead to toxicity, the very principles upon which Cytokinetics was started. Mava rapidly proved to be a remarkably interesting potential drug, and understanding how Mava reduced the power output of the heart by binding to cardiac myosin became of great interest. Eric Green joined MyoKardia as Head of Translational Research in early 2014 to lead efforts to understand how Mava works. Eric received clinical training in internal medicine and cardiology at Brigham and Women’s Hospital. Prior to joining MyoKardia, he served as Co-founder and Scientific Adviser of iLab Solutions, Clinical Research Director of Element Sciences, and Co-founder and CEO of Respira Design. As mentioned in the Introduction, in a study using the Seidmans’ HCM mouse models, Eric collaborated with Kricket and Jon Seidman to show that Mava decreases cardiac contractility, suppresses the onset of hypertrophy and cardiomyocyte disarray when mice were treated early with the potential drug, and even reverses these HCM-induced changes when the mice were treated after the changes had occurred (Green et al., 2016).

These mouse model results were highly encouraging, but as I indicated earlier, it is not always easy to translate results from mice to man. Furthermore, the foundational molecular basis on which we started the company came into question when our work at Stanford began to reveal unexpected results from our studies on the effects of myosin HCM mutations on the fundamental parameters of myosin – velocity (v) measured with our *in vitro* motility assay, intrinsic force (F_intrinsic_) measured with our dual-beam laser trap assay, and the rate of the actin-activated myosin ATPase (k_cat_). We were expecting that all the HCM mutations in myosin would elevate one or more of these three parameters and that would be the basis of the mutated myosin’s expected hypercontractility. However, measurement for multiple HCM mutations indicated that while one parameter was increased for a particular mutation, other parameters for that mutation were often decreased, making it difficult to see how the ensemble of effects could lead to hypercontractility (Spudich et al., 2024). Furthermore, in two cases, R663H and G741R, no changes were seen in any of these three parameters compared to wild type human β-cardiac myosin (Kawana et al., 2017a; Sarkar et al., 2020; Spudich et al., 2024). These results were not only unexpected but perplexing and *put into question whether the myosin HCM mutations caused myosin itself to generate a higher power output*.

Then a dream I had (see https://www.ibiology.org/cell-biology/muscle-biology/#part-4) inspired me to propose a unifying hypothesis that some sarcomeric protein with a domain that is overall negatively charged could be interacting with a positively charged flat surface of the cardiac myosin (which I named the myosin mesa) keeping a subpopulation of the cardiac myosin in an *OFF-state*, unable to interact with actin (Spudich, 2015). Such heads could be held in reserve and released by appropriate signaling mechanisms when higher power output from the heart was needed. I proposed a unifying hypothesis that most, if not all, myosin HCM mutations are weakening the protein-protein interactions in this *OFF-state*, increasing the number of heads accessible for interacting with actin (N_a_) and thereby causing the hypercontractility seen in HCM patients (Spudich, 2015). I also proposed that MyBP-C might interact with the myosin mesa and that dysregulation of MyBP-C, like the HCM mutations in myosin, leads to increases in N_a_, *accounting for why the vast majority of HCM mutations occur in the two sarcomere proteins, β-cardiac myosin and MyBP-C*.

## The proximal tail domain of myosin is involved in a sequestered *OFF-state* known as the Interacting Heads Motif (IHM)

Figure 2A shows a schematic version of myosin with a truncated S2 region, showing just the proximal part of the S2 coiled-coil tail that consists of the first ∼15 heptads. These S1 heads are shown in an *ON-state*, able to interact with actin. Myosin exists in an *OFF-state* that involves the folding back of the S1 heads onto the molecule’s own proximal S2, with the two heads also interacting with one another asymmetrically in what is known as the Interacting Head Motif (IHM) (Figure 2B) (Wendt et al., 2001; Woodhead et al., 2005; Burgess et al., 2007; Lowey and Trybus, 2010; Woodhead et al., 2013; Al-Khayat et al., 2013; Lee et al., 2018; Craig and Padron, 2022; Auguin et al., 2024). A *partial OFF-state* is also thought to exist in which the S1 head that is not interacting significantly with the proximal S2 is free to interact with actin (Figure 2C). Homology models of the cardiac myosin IHM state have been used to study the effects of HCM mutations on this state (Nag et al., 2017; Alamo et al., 2017; Robert-Paganin et al., 2018), and we showed that the myosin mesa is involved in the interacting domains of this IHM state (Figure 2B) (Nag et al., 2017). Homology models, however, are only models, and obtaining a high-resolution structure of the human β-cardiac myosin IHM state was a high priority for several years. We teamed up with Anne Houdusse at the Curie Institute in Paris to achieve this goal.

In 2023, Anne Houdusse and her colleagues used our purified myosin and high-resolution electron microscopy to obtain a 3.6 Å resolution structure of human β-cardiac myosin IHM (PDB 8ACT) (Grinzato et al., 2023). Not unexpectedly, there are significant differences between the high-resolution EM structure and the homology models used previously (Grinzato et al., 2023; Lee et al., 2024). The high-resolution structure is the only IHM structure to provide atomic details needed to establish which conformation the heads must adopt to form the asymmetric IHM configuration, or to say anything about positions of side chains and how they might interact. But, as we had described previously using one of the homology models (Nag et al., 2017), the high-resolution structure confirms that the myosin mesa is an important interface in stabilizing the IHM *OFF-state* (Figure 2B) (Grinzato et al., 2023; Spudich et al., 2024).

A direct biochemical assay for measuring whether an HCM mutation causes the release of more myosin heads for interaction with actin utilizes two different 2-headed constructs of human β-cardiac myosin – one with a short coiled-coil proximal tail (8-heptad or shorter), which cannot form the IHM state, and the other with a longer tail (15-heptad or longer), which can form the IHM state. The actin-activated ATPase activity of the long-tail construct is typically 40%–50% lower than its corresponding short-tail construct, presumably because the long-tail construct can form the IHM *OFF-state*. The 40%–50% *OFF-state* heads could be in molecules that are in the IHM *OFF-state* (Figure 2B), in a *partial OFF-state* (Figure 2C), or some mixture of the two. More research is needed to determine to what extent *partial OFF-state* molecules play a role.

If an HCM mutation destabilizes the IHM state in these biochemical measurements, then the long-tail constructs will approach the ATPase values of the short-tail constructs. We call this the long-tail/short-tail ATPase ratio (LSAR) assay. Out of 20 mutations in human β-cardiac myosin to which the LSAR assay has been applied, all but one shows an increase in additional N_a_ by the LSAR assay (Spudich et al., 2024), consistent with the ‘unifying hypothesis’ put forward 9 years ago (Spudich, 2015). Importantly, while 6 of these are at interfaces stabilizing the IHM, 13 are elsewhere in the structure and de-stabilize the IHM allosterically. Thus, one cannot predict the effect of an HCM mutation on the stability of IHM by simply examining the structure. The changes seen in k_cat_, velocity and intrinsic force for sure modify the effect of de-stabilizing the IHM, sometimes enhancing the increase in power output and sometimes adding a hypocontractility contribution (Spudich et al., 2024).

## Mavacamten binds directly to β-cardiac myosin and lowers the power output of the heart

**FIGURE 5:**
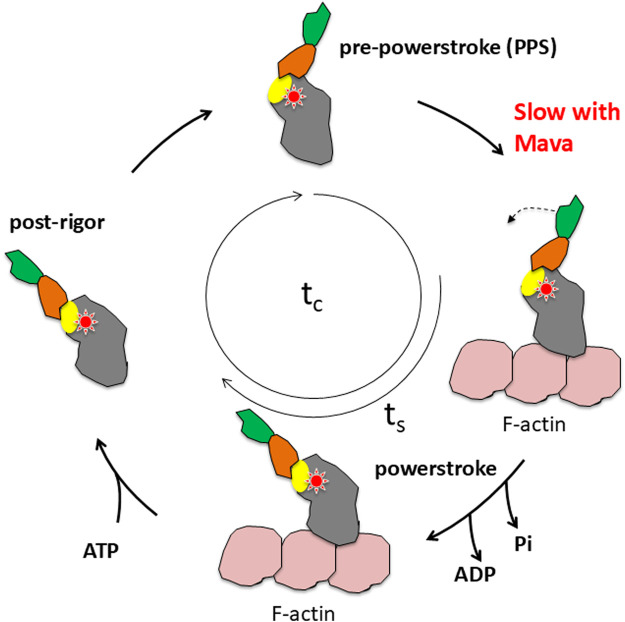
Schematic view of the actin-activated myosin ATPase cycle. The red star indicates the approximate fulcrum point about which the lever arm swings and is also the approximate site of Mava binding. The yellow oval indicates the position of the converter, which is the first part of the swinging lever arm. The light-chain binding region (orange and green) amplifies the movement of the converter. The total cycle time is t_c_ and the time strongly bound to actin is t_s_.

In molecular studies at MyoKardia, the basic research team showed that Mava acts on β-cardiac myosin S1 to decrease its actin-activated ATPase activity, Pi release rate, and affinity for actin (Figure 5) (Kawas et al., 2017), all of which would reduce contractility of the heart. A second effect of Mava might be to stabilize the IHM *OFF-state*. A possible biochemical correlate of the structural IHM state is an innovative biochemical assay developed by Roger Cooke and his colleagues, who demonstrated in both skeletal and cardiac fibers an *OFF-state* of myosin known as the super relaxed state (SRX), identified by a 10-fold reduction in myosin basal ATPase (Hooijman et al., 2011). Two studies with purified β-cardiac myosin showed that Mava drives 100% of myosin heads into the SRX state, one from Dave Thomas’ laboratory (Rohde et al., 2018) and the other from a collaboration between my Stanford laboratory and MyoKardia (Anderson et al., 2018). In the Anderson et al. study, we reported an increase in the number of compacted heads in the presence of Mava (Anderson et al., 2018), which might or might not represent the IHM state (Nag et al., 2023; Spudich et al., 2024). Mava-induced sequestering of heads into a folded compact state could add to a decreased contractility of the heart.

**FIGURE 6:**
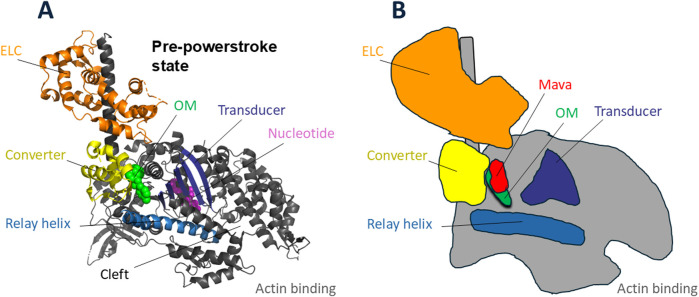
Cardiac myosin head with sites of omecamtiv mecarbil (OM) and mavacamten (Mava) binding. **(A)** Pre-powerstroke structure of cardiac myosin head domain [PDB code 5N69 (Planelles-Herrero et al., 2017)] with OM (green) bound at the base of the converter (yellow), essentially at the fulcrum point of rotation of the lever arm. The binding pocket is nestled between the converter, the relay helix (blue), and the transducer (dark blue). The nucleotide (purple) active site is more central in the globular head. **(B)** Schematic drawing of the OM-bound structure in **(A)** showing the position of Mava (red) in relation to OM.

As always, obtaining high-resolution structural information of an enzyme of interest, with and without drug bound, is essential to understand how that drug is modifying the catalytic properties of that enzyme. Two important recent papers from the laboratories of Roger Craig and Raul Padron (Dutta et al., 2023) and Stefan Raunser (Tamborrini et al., 2023) described cryo-EM structures of the cardiac thick filament in the presence of Mava at resolutions of ∼6 Å and ∼8 Å, respectively. These papers offer the best available information about the interaction between the different protein components of the thick filament in the presence of Mava, including the role of MyBP-C, which I describe as a possible key regulatory element in formation of the *OFF-state* of myosin in my unifying hypothesis (Spudich, 2015; Spudich et al., 2024). The resolution of those structures, however, was not sufficient to see where Mava bound or how Mava influences the catalytic and structural properties of the cardiac myosin. The Houdusse laboratory has solved, at resolutions better than 2.8 Å, the structures of β-cardiac myosin single headed constructs complexed with Mava (Auguin et al., 2024), and she and her colleagues solved the structure of the cardiac myosin motor domain previously with OM bound (Planelles-Herrero et al., 2017). Mava and OM both co-crystallize with the myosin constructs in a pre-powerstroke state, a state that traps the hydrolysis products with a lever arm in its prestroke configuration. Remarkably, Mava and OM bind to the same general pocket near the converter domain of myosin (Auguin et al., 2024) (Figure 6), OM causing activation of contractility and Mava causing a reduction in contractility. Houdusse and her colleagues combined their structural analyses with molecular dynamics to explain how the two small molecules, occupying the same pocket, result in opposite effects on contractility (Auguin et al., 2024).

OM’s mechanisms of action leading it to be an activator are multifaceted. First, OM binding increases the actin-dependent Pi release rate (Hartman et al., 2011), the rate limiting step in the actin-activated ATPase cycle (Figure 5). It binds in a pocket near the converter of the motor domain (Planelles-Herrero et al., 2017) (Figure 6), and it interacts with the Relay helix, exerting restraints on its position. This restraint stabilizes the lever arm in the pre-powerstroke state position (Auguin et al., 2024). Importantly, OM-bound myosin favors states with a prestroke lever arm that are also able to interact with actin. When OM is bound, the powerstroke is suppressed and actin binding is prolonged, which slows the velocity of actin sliding along cardiac myosin heads (Aksel et al., 2015; Liu et al., 2015; Swenson et al., 2017; Liu et al., 2018). This prolonged binding activates the tropomyosin-troponin Ca^2+^ regulatory system (Woody et al., 2018; Spudich, 2019; Nakanishi et al., 2022), providing increased force and power output. Furthermore, OM destabilizes the IHM *OFF-state* of myosin (Auguin et al., 2024) making more heads available for interacting with myosin, further increasing power output.

Mava’s mechanisms of action leading it to be an inhibitor are also multifaceted. Mava binding also maintains the lever arm in the prestroke position, but it shows different dynamics in the pocket. The bulkier Mava molecule is accommodated by a wider pocket than seen with OM. Whereas the longer OM interacts with the Relay Helix, Mava is too short to do so (Figure 6). OM and Mava both interact with the converter (Figure 6), but the side chain interactions are different, and differences in the converter orientations are apparent. None of these changes are readily apparent in lower-resolution images. The structures illustrated in Figure 6 are the OM-bound structure (Planelles-Herrero et al., 2017). The schematic shown in Figure 6B is not very different for the Mava-bound structure, and one needs to compare the OM-bound and Mava-bound high-resolution structures to fully appreciate the important differences that make one an activator and the other an inhibitor. The reader is referred to Auguin, Robert-Paganin et al. who describe the differences in detail (Auguin et al., 2024). The bottom line is that Mava-bound myosin cannot release Pi readily and the drug-bound pre-powerstroke state interacts poorly with actin, causing myosin heads to be effectively removed from the actin-activated ATPase cycle, resulting in a decrease of power output. Mava bound heads, unlike OM-bound ones, are compatible with the IHM conformation. Whether Mava stabilizes the IHM state is an open question, but if so, pulling heads out of the cycle by putting them in an IHM *OFF-state* would further decrease N_a_ and reduce power output in the heart.

## Conclusion and perspectives

Mavacamten, the first in class drug for treatment of HCM, is a success story deserving of the Galien prize. Its success depended on detailed understanding of the molecular mechanism of myosin function. Also critical to its success was a host of individuals performing their critical roles at every step along the path of taking this drug to market – the Founders of MyoKardia, the CEO and other chief executive officers, the entire team at MyoKardia, the Board of Directors, Charles Homcy and Third Rock Ventures, big pharma partners, clinicians around the world who carried out the clinical trials, the patients who signed up for those trials, and the list goes on. However, many other drug discovery efforts with a similar cadre of talented individuals fail. Those failures occur for a variety of reasons, but I believe high on that list is a failure to choose a target that is mechanistically understood as thoroughly as the contractile proteins of the heart. The fact that they are downstream effectors, which avoids pleiotropic effects on muscle cells that are otherwise often encountered, also plays a role. But it is the deep understanding of the biochemistry, biophysics, and physiology of the contractile system, and of the molecular motor myosin particularly, that increased the probability of success in this therapeutic pursuit.

This same deep understanding is also leading to aficamten, a second-in-class human β-cardiac myosin inhibitor being developed by Cytokinetics. Aficamten is in Phase 3 clinical trials (Maron et al., 2024) and promises to be another excellent treatment for HCM, with somewhat different properties from Mava. This is an exciting development. No drug is perfect and being able to choose between more than one drug for the treatment of any disease is always highly desirable. Interestingly, aficamten binds to a different pocket in the S1 globular head than Mava and OM (Hartman et al., 2024).

I want to emphasize how important networking is to the success of both basic science and biotech endeavors. In basic science, most efforts require an interdisciplinary approach. Assuming you have a diverse early training, your interdisciplinary work can be implemented by recruiting students, postdocs, sabbatical visitors and others to your lab from different areas, such as biochemistry, physics, structural biology and genetics, and create a local network of diversely trained people working together to solve a pivotal research problem of the time. This is the primary route I have taken in my career. It has the advantage that you are in constant contact with everyone in the network, helping them to interact with one another in a catalytic manner, which leads to creative research as well as all of them obtaining interdisciplinary training. On occasion, collaborations with other laboratories are an efficient way to incorporate new approaches into your lab’s efforts. In more general terms, networking with the scientific community at large often generates new ideas for approaches to solve your biological question. But most of your time should be focused on your own lab, thinking about the experiments being done day and night and even dreaming about them! Creative research is hard, and not for the less than fully committed investigator. Another critical aspect is being very good at identifying and recruiting the right people into your lab, and the right people to collaborate with. Keep your lab small but filled with carefully selected members, and treat them as your scientific family, nurturing them and allowing them to grow in their own way.

It might seem daunting to simultaneously manage an academic lab and start a biotech effort, but it can be done. The answer is to focus. In your basic science lab, choose an important research area that is poorly understood and that excites you, then focus on the pivotal experiments needed to generate the understanding of the functions of interest. Do not waste time doing experiments just because you can, a trap easily fallen into. Go for the experiments that will truly answer the fundamental question being pursued. If you need to develop new technologies to get to your answer, do not be afraid to do so. Also, focus on gathering the right people together to accomplish your goals. In your basic science lab, that means your students, postdocs, and others. Who you choose to join will determine the trajectory of your effort. When it comes to translating your work to clinically relevant issues, networking and choosing who your partners will be becomes more complicated, because you need a much wider variety of colleagues and partnerships. If you are going after a small molecule therapeutic, you will rapidly need a number of chemists, pharmacologists, clinically trained folks, and, from the beginning, someone needs to be thinking about the business side of the company. Just as for your academic lab, who joins your new biotech company is critical. But the good news is that once a few key members are chosen, including a CEO, who has the major responsibility of running the company, you can settle into a scientific advisory role as a Founder of the company and not be involved in day-to-day matters. The secret is identifying the right people – it’s always about that. If you set things up correctly from the start, your time commitment to your company can easily be a day a week or less. This is the path I took with both Cytokinetics and MyoKardia, as I have attempted to illustrate in this article, describing in some detail the key people who were brought on board and their individual talents, expertise and prior experiences.

In conclusion, I am pleased to have been one of the Co-Founders along with Ron Vale, Larry Goldstein, and James Sabry to first translate molecular motor research into the world of biotechnology by founding Cytokinetics in 1998. And it has been gratifying to see the expansion of molecular motor therapeutics in other biotech companies as well as in big pharma companies, with Cytokinetics having paved the way. Kainomyx, Inc., for example, which I co-founded in 2019 together with Darshan Trivedi, Suman Nag, Kathleen Ruppel, and Anna Spudich, is targeting the contractile proteins of various parasites to help eliminate devastating parasitic diseases around the world, including malaria, which kills one child under the age of five every 2 minutes while you are reading this article. Kainomyx is taking advantage of the essentiality of contractile proteins, including myosin, in all parasitic cells to develop novel therapeutics for these devastating diseases.
