# Supplementary material for: From amoeboid myosin to unique targeted medicines for a genetic cardiac disease
Source: Front Physiol. 2024 Oct 28;15:1496569. doi: 10.3389/fphys.2024.1496569 (PMC11550953; doi:10.3389/fphys.2024.1496569)
Supplement: Supplementary file 2 [file Presentation1.pdf]

frontiers | Frontiers in Physiology

Supplemental Information

From amoeboid myosin to unique targeted medicines for a genetic cardiac disease

James A. Spudich

Department of Biochemistry, Stanford University School of Medicine, Stanford, CA, United States

Supplementary Movie 1. A *Dictyostelium* myosin II-null cell expressing GFP-myosin II shows localization of the myosin-II to the cleavage furrow during cell division. The cell is ~5 μm in diameter. For details, see (Zang and Spudich 1998).

Supplementary Movie 2.

Me4 rhodamine-phalloidin-labeled actin filaments observed moving by fluorescence microscopy along a lawn of myosin molecules attached to a glass surface in the presence of ATP (Kron and Spudich 1986) (Toyoshima, Kron et al. 1987).

Kron, S. J. and J. A. Spudich (1986). "Fluorescent actin filaments move on myosin fixed to a glass surface." Proc Natl Acad Sci U S A **83**(17): 6272-6276.

Toyoshima, Y. Y., S. J. Kron, E. M. McNally, K. R. Niebling, C. Toyoshima and J. A. Spudich (1987). "Myosin subfragment-1 is sufficient to move actin filaments in vitro." Nature **328**(6130): 536-539.

Zang, J. H. and J. A. Spudich (1998). "Myosin II localization during cytokinesis occurs by a mechanism that does not require its motor domain." Proc Natl Acad Sci U S A **95**(23): 13652-13657.
